# Protein analysis and gene expression indicate differential vulnerability of Iberian fish species under a climate change scenario

**DOI:** 10.1371/journal.pone.0181325

**Published:** 2017-07-18

**Authors:** Tiago F. Jesus, João M. Moreno, Tiago Repolho, Alekos Athanasiadis, Rui Rosa, Vera M. F. Almeida-Val, Maria M. Coelho

**Affiliations:** 1 Centro de Ecologia Evolução e Alterações Ambientais, Faculdade de Ciências, Universidade de Lisboa, Lisboa, Portugal; 2 Laboratório Marítimo da Guia, MARE—Centro de Ciências do Mar e do Ambiente, Faculdade de Ciências da Universidade de Lisboa, Av. Nossa Senhora do Cabo, Cascais, Portugal; 3 Instituto Gulbenkian de Ciência, Rua da Quinta Grande, Oeiras, Portugal; 4 Laboratório de Ecofisiologia e Evolução Molecular, Instituto Nacional de Pesquisas da Amazônia (INPA), Manaus, AM, Brasil; Fred Hutchinson Cancer Research Center, UNITED STATES

## Abstract

Current knowledge on the biological responses of freshwater fish under projected scenarios of climate change remains limited. Here, we examine differences in the protein configuration of two endemic Iberian freshwater fish species, *Squalius carolitertii* and the critically endangered *S*. *torgalensis* that inhabit in the Atlantic-type northern and in the Mediterranean-type southwestern regions, respectively. We performed protein structure modeling of fourteen genes linked to protein folding, energy metabolism, circadian rhythms and immune responses. Structural differences in proteins between the two species were found for HSC70, FKBP52, HIF1α and GPB1. For *S*. *torgalensis*, besides structural differences, we found higher thermostability for two proteins (HSP90 and GBP1), which can be advantageous in a warmer environment. Additionally, we investigated how these species might respond to projected scenarios of 3° climate change warming, acidification (ΔpH = -0.4), and their combined effects. Significant changes in gene expression were observed in response to all treatments, particularly under the combined warming and acidification. While *S*. *carolitertii* presented changes in gene expression for multiple proteins related to folding (*hsp90aa1*, *hsc70*, *fkbp4* and *stip1*), only one such gene was altered in *S*. *torgalensis* (*stip1*). However, *S*. *torgalensis* showed a greater capacity for energy production under both the acidification and combined scenarios by increasing *cs* gene expression and maintaining *ldha* gene expression in muscle. Overall, these findings suggest that *S*. *torgalensis* is better prepared to cope with projected climate change. Worryingly, under the simulated scenarios, disturbances to circadian rhythm and immune system genes (*cry1aa*, *per1a* and *gbp1*) raise concerns for the persistence of both species, highlighting the need to consider multi-stressor effects when evaluating climate change impacts upon fish. This work also highlights that assessments of the potential of endangered freshwater species to cope with environmental change are crucial to help decision-makers adopt future conservation strategies.

## Introduction

Climate change is threatening biodiversity worldwide, with temperature and atmospheric CO_2_ values rising at an unprecedented rate [1–3]. Shifts in thermal, precipitation and flow regimes will be particularly harmful for freshwater ecosystems [1]. Increases in water temperature, coupled with decreased river flow and increased severity and frequency of droughts, will undoubtedly pose new challenges for freshwater fauna, particularly in the Mediterranean region [4]. Such changes in natural freshwater ecosystems, will directly influence the survival, and ultimately the persistence, of extant species.

In order to cope with future climate changes, species can shift their distribution to a more suitable habitat, change their life-cycle or adapt through micro-evolution or plasticity to new environmental conditions [5]. Otherwise they may become extinct [5]. Fish metabolism strongly depends on the environmental temperature [6], and freshwater fish often have limited ability to migrate to a more suitable river, making them vulnerable to environmental changes [7]. Evidence of coping mechanisms for climate change are emerging for teleost fish species such as chinook and sockeye salmon (*Oncorhynchus tshawytscha* and *O*. *nerka*), in which both new migration patterns and plasticity in thermal tolerance have been observed [8,9]. Also, the reef fish *Acanthochromis polyacanthus* and the rainbowfish *Melanotaenia duboulayi* have exhibited changes in gene expression in response to warming, both through plasticity mechanisms and processes that may enable them to adjust over generations [10,11].

European climate change reports highlight the importance of an ongoing process that has already diminished river flow and increased mean water temperature between 1 and 3°C, over recent decades [1–4]. These issues are noticeable for many European rivers during the summer season and particularly for southern European rivers where the severity and frequency of droughts has significantly increased [4].

The Iberian Peninsula is at the frontier between two contrasting climate types: the Atlantic in the northern region that is characterized by mild temperatures, and the Mediterranean in the southern region (one of 25 biodiversity hotspots [12]), typified by high temperatures and droughts [13–16]. Freshwater fish of the *Squalius* genus (Cyprinidae family) are endemic to river basins and regions in these two different climates, providing an opportunity to study closely-related species under these two climate types [17]. *S*. *carolitertii* [18] inhabits the Atlantic-type northern region, whereas *S*. *torgalensis* [19], a critically endangered species, has a more restricted distribution within the Mira river basin in the Mediterranean-type southwestern region [19]. Hence, these two species reside under different environmental conditions, with distinct seasonal and even daily water temperature fluctuations, and demonstrate different traits that are possibly the result of adaptation to these contrasting environmental conditions [14,15]. Compared to *S*. *carolitertii*, S. *torgalensis* has a shorter life span, earlier spawning age, and a smaller body size, all of which are characteristics of species inhabiting more unstable environments [15]. Also, *S*. *torgalensis* may be better adapted to cope with higher temperatures, since it is able to induce *hsps* genes in response to high temperatures and acute thermal stress [14,20]. Conversely, *S*. *carolitertii* was shown to be unable to cope with temperatures as high as 35°C and either lacked or presented a weak response in terms of *hsps* gene expression under stress [14–20]. Furthermore, in a transcriptomic study, these two species presented differences in gene expression patterns between control (18°C) and heat shock treatment (30°C) [20]. Moreover, a vast set of potential target genes involved in protein folding, energy metabolism, circadian rhythms and immune responses for use in thermal studies of these species has become available.

Climate change threatens to significantly impact the survival and persistence of fish, particularly for species living close to their thermal tolerance limits and are thus prone to be harmed by small changes in environmental temperatures [21–27]. In this sense, adaptation of these species to their current environmental conditions may provide important clues as to how they might endure future environmental changes. Besides rising temperatures, acidification can also affect freshwater biota [28]. Recently, considerable attention has been given to ocean acidification and this process is widely known to affect the physiology and behavior of many marine species (e.g. [29–34]), ranging from changes in olfactory systems [29], neurotransmitter malfunctions [35] and skeletal deformities [36,37]. Unlike ocean acidification which is caused by elevated atmospheric CO_2_ concentrations, lake and river acidification is mainly driven by acid rain [38]. However, freshwater acidification is also likely to be affected by future increases in CO_2_ levels [39]. To date, few studies have examined the effects of increasing CO_2_ and acidification, as mediated by climate change, on freshwater fish [40].

Here, we aim to understand how freshwater fish might respond to projected future climate change scenarios of warming and acidification and their combined effects. We studied two Iberian endemic fish, *S*. *carolitertii* and *S*. *torgalensis*. Both species have distinct evolutionary backgrounds and experience differing environmental conditions. We simulated a climate change scenario for the year 2100, consisting of a summer average temperature increase of 3°C and a ΔpH = -0.4. Therefore, we based our parameters on the IPCC Representative Concentration Pathways (RPC 8.5) from the fifth Assessment Report (AR5) [1, 41], since it projects an increase of air temperature ranging from 2.6 to 4.8 and an increase in oceanic water acidification of ΔpH = -0.42. In this context, we investigated fourteen genes linked to warming and/or water acidification responses in fish, taking advantage of their differential expression in the transcriptomes of *S*. *carolitertii* and *S*. *torgalensis* [20]. Specifically, we used genes involved in protein folding, energy metabolism, circadian rhythms and immune responses in order to: i) compare the differences between the two species protein structural and functional configurations, and ii) assess alterations in gene expression between control and experimental conditions. Integration of our results allowed us to evaluate the potential capacity of the endemic freshwater fish to cope with future climate change scenarios.

## Methods

### Sampling

Twenty-four wild adult fish of *S*. *carolitertii* and *S*. *torgalensis* species were collected from Portuguese rivers, Mondego (40° 8'5.22"N; 8° 8'35.06"W) and Mira (37°38'1.31"N; 8°37'22.37"W), respectively, by electro-fishing (300V, 4A). Short duration pulses were used in order to avoid juvenile mortality. Sampling was performed during spring season (when average water temperatures were 17.8 +- 0.67°C for Mondego river and 19.5 +- 0.21°C for Mira river and average water pH were 8.08 +- 0.01 for Mondego river and 8.23 +- 0.02 for Mira river). Fish were captured under a license (263/2014/CAPT) issued by Portuguese authority for Conservation of endangered species (ICNF [Instituto da Conservação da Natureza e das Florestas]).

### Experimental design

Upon arrival to the aquaculture facilities of Laboratório Marítimo da Guia (Faculdade de Ciências da Universidade de Lisboa, Portugal) fish were placed in tanks with conditions (temperature, pH and conductivity) similar to the ones found in nature during sampling. Then, fish were slowly acclimated to the control experimental conditions, in eight 200 L tanks (four per species), for 2 weeks, mimicking summer average values for temperature (18,68 +- 0.38 for *S*. *carolitertii* and 23.02 +- 0.77 for *S*. *torgalensis*) and pH (6.88 +- 0.33 for *S*. *carolitertii* and 7.31 +- 0.51 for *S*. *torgalensis*), under normoxic (8 mg/L) conditions (control condition, see Table 1).

**Table 1:** Experimental conditions performed for both species. Control conditions defined for each species was based on summer average water temperature and pH [data obtained from snirh.pt (National Information System of Water Resources) for 4 consecutive years (2001–2005)]. Test conditions consist of an increase of 3°C in relation to the current summer average conditions (Warming and Combined) and a decrease of 0.4 units in the current summer pH average (Acidification and Combined).

Species	Condition	Temperature	pH
*S*. *carolitertii*	control	19°C	6.9
	warming	22°C	6.9
	hypercapnia	19°C	6.5
	combined	22°C	6.5
*S*. *torgalensis*	control	23°C	7.3
	warming	26°C	7.3
	hypercapnia	23°C	6.9
	combined	26°C	6.9

After laboratory acclimation, four different groups (with 5 to 7 individuals) of *S*. *carolitertii* and *S*. *torgalensis* were gradually acclimated to four different conditions (Table 1): i) control; ii) warming; iii) acidification and iv) combined warming and acidification condition. Within these experimental conditions, we planned to simulate a moderate climate change scenario by increasing the temperature in +3°C and applying a ΔpH = -0.4, under a 2x2 factorial design. During the acclimation and experimental periods, fish were fed daily (ad libitum) with bloodworms (TMC Iberia, Portugal), white mosquito larvae (TMC Iberia, Portugal) and *Spirulina* spp. flake food (Ocean nutrition, Belgium). Overhead tank illumination was provided, according to prevailing natural light conditions, under a 12:12 (day: night) light regime. Ammonia, nitrite and nitrate levels were monitored daily (Salifert Profi Test, Holland) and kept always below detectable levels. Normoxic conditions were maintained and pH values were monitored and adjusted automatically by means of a computerized controlling system (Profilux 3.1N, GHL, Germany) connected to individual oxygen and pH probes (GHL, Germany), respectively. Monitoring was performed every 2 seconds and pH values were adjusted through injection of N_2_/CO_2_ (Air Liquide, Portugal) and upregulated by aeration with CO_2_ filtered air (soda lime, Sigma-Aldrich). Conductivity was individually monitored (Profilux 3.1N, GHL, Germany) and kept between 400–500 μS/cm. Automatic dosing systems (TMC Iberia, Portugal), linked to the Profilux system, enabled inflow of freshwater (300 or 600 μS/cm), in order to lower or raise conductivity values (culture tanks), within desired interval (400–500 μS/cm). After 30 days of experimental exposure, five to seven individuals of each treatment and species were euthanized (with spinal transection followed by immediate brain removal), during early morning period. Experimental procedures used in this research were in accordance with the requirements imposed by the Directive 2010/63/EU of the European Parliament and of the Council of 22 September 2010 on the protection of animals used for scientific purposes (reviewed and approved by the animal ethics committee ORBEA–Animal Welfare Body of FCUL Statement 5/2016).

### RNA extraction and cDNA synthesis

Liver and muscle tissue samples were immediately collected from fish and stored using RNAlater (Ambion, Austin, TX, USA), following the manufacturer’s instructions. For ribonucleic acid (RNA) extraction, TRI Reagent (Ambion, Austin, TX, USA) was added to liver and muscle samples. After homogenization with a Tissue Ruptor (Qiagen, Valencia, CA, USA), RNA was extracted according to the TRI Reagent manufacturers protocol. TURBO DNase (Ambion, Austin, TX, USA) was employed to degrade any remaining genomic contaminants, followed by phenol/chloroform purification and LiCl precipitation [42]. Sample quality was checked using a Nanodrop-1000 spectrophotometer (Thermo Scientific, Waltham, MA, USA) based on the 260/280 nm and 260/230 nm absorbance ratios. Sample concentration were determined to ensure sufficient quantity of homogeneous RNA for complementary DNA (cDNA) synthesis. Synthesis of cDNA was performed, according to manufacturer’s instructions, using a RevertAid H Minus First Strand cDNA synthesis kit (Thermo Fisher Scientific, Waltham, MA, USA) and stored subsequently at -20°C.

### Target genes

A total of fourteen genes of interest were chosen among the differentially expressed genes, belonging to different biological functions (protein folding, energy metabolism, circadian rhythm and immune response) (detailed in Table 2), in the transcriptomes of *S*. *carolitertii* and *S*. *torgalensis* [20].

**Table 2:** List of target genes, with their official gene names, gene descriptions and functional category.

gene name	gene description	Function	Functional category
*hsc70*	heat shock cognate 70	Folding of denatured proteins; protects cells from stress.	protein folding
*hsp70*	heat shock protein 70	Folding of denatured proteins; protects cells from stress.	protein folding
*hsp90*	heat shock protein 90	Folding of denatured proteins; protects cells from stress.	protein folding
*stip1*	stress-induced phosphoprotein 1	links HSP70 and HSP90 together.	protein folding
*fkbp4*	FK506 binding protein 4	This gene is involved in immunoregulation and basic cellular processes involving protein folding and trafficking.	protein folding
*hif1a*	hypoxia inducible factor 1 alpha	It induces several genes involved in hypoxia response, cell proliferation, glucose and iron metabolism.	energy metabolism
*ldha*	lactate dehydrogenase A	Catalyzes the inter-conversion of pyruvate and L-lactate	energy metabolism
*cs*	citrate synthase	Catalyzes the first reaction of the citric acid cycle: the condensation of acetyl-CoA and oxaloacetate to form citrate.	energy metabolism
*ndufb8*	mitocondrial NADH dehydrogenase (ubiquinone) 1 beta subcomplex subunit 8	Accessory subunit of the NADH dehydrogenase (ubiquinone) complex, located in the mitochondrial inner membrane, of the electron transport.chain.It transfers electrons from NADH to the respiratory chain.	energy metabolism
*glula*	glutamate-ammonia ligase (glutamine synthase) a	Catalyzes the condensation of glutamate and ammonia to form glutamine.	energy metabolism
*lox*	lysyl oxidase	Catalyzes the formation of aldehydes from lysine residues in collagen and elastin precursors.	energy metabolism
*per1a*	period circadian clock 1a	It is a member of the period gene family and is important for circadian clock maintenance.	circadian rhythm
*cry1aa*	cryptochrome 1a	It is a member of the cryptochrome gene family, which regulated the circadian clock in a light dependent fashion.	circadian rhythm
*gbp1*	guanylate binding protein 1	This gene is induced by interferons and presents antiviral activity by regulating the inhibition of proliferation of endothelial cells.	immune response

For both species, the sequences of the target genes were obtained from Genomic Resources Development Consortium *et al*. 2015 [43]. All pairs of primers used were designed using PerlPrimer software v.1.1.19 [44] (S1 and S2 Tables, supporting information). Sequences that displayed polymorphisms between both species were re-sequenced by Sanger (S1 Table, supporting information). CLC Sequence Viewer v7.5 (CLC bio, Aarhus, Denmark) was employed to align nucleotide sequences. Complete sequences were obtained, except for *per1a* gene for which transcriptome information only permitted to study the partial coding sequence. The obtained sequences were deposited in GenBank (Accession numbers: KX589462-KX589485). Nucleotide sequences were translated and the resulting protein sequences were aligned using CLC Sequence Viewer v7.5 (CLC bio, Aarhus, Denmark) under default parameters (gap open cost: 10; gap extension cost: 1; end gap cost: as any other; and alignment method very accurate).

### Protein structure prediction

In order to predict physical and chemical parameters, the ProtParam tool [45] was used. Protein three-dimensional structure was also predicted using the homology modelling algorithm (RaptorX Structure Prediction) offered in RaptorX webserver [46]. Protein structural alignments of each species were performed following the Smith-Waterman algorithm offered in UCSF Chimera [47], using the default parameters with a secondary structure score set to 0.70. Protein alignments were performed using the same protein of each species and differences are presented, with differing amino acid residues highlighted.

### Quantitative RT-PCR

Relative expression levels of genes of interest were normalized against three reference genes [Poly(A) binding protein, cytoplasmic 1a (*pabpc1a*), ribosomal protein L35 (*rpl35*) and ribosomal protein SA (*rpsa*)] (for details on primer conditions see S2 Table, supporting information), chosen among the most stable genes for the transcriptomes of three organs (liver, fins and skeletal muscle) of these two species exposed to different temperature conditions (18°C and 30°C) [43]. These reference genes were chosen from contigs with more than 1000 read counts per library, FDR > 0.05 and Fold Change < 1.5 (log_2_(Fold Change) < 0.58), in order to assure that they are highly expressed, but not differentially expressed. Furthermore, reference genes stability was also verified in *Squalius pyrenaicus* transcriptome [48], to further guarantee their stability across more conditions (S3 Table, supporting information). In order to determine the stability of these reference genes, in the qPCR analysis, we used the NormFinder software [49].

Real-time polymerase chain (PCR) reactions were performed in a Bio-Rad CFX96 system (Bio-Rad, USA), following manufacturer’s instructions for Sso Advanced universal SYBR® Green supermix (Bio- Rad, Hercules, CA, USA). Controls without template and without reverse transcriptase were included to check for PCR contamination and genomic deoxyribonucleic acid (DNA) contamination, respectively. Amplicons identities were confirmed through melting curve analysis. The PCR efficiency for each sample was assessed using LinRegPCR 11.1 software [50] and ranged from 94.38%–97.72% for all primer pairs (S2 Table, supporting information). Relative quantity of genes of interest was calculated, using the comparative threshold cycle (CT) method with efficiency correction, using the mean PCR efficiency for each amplicon [50].

Relative gene expression of target genes was calculated against the geometric mean of the reference genes, using the 2-(ΔΔCt) method [51].

Data was log transformed [log10(x+1)] and checked for normality (Shapiro-Wilk’s test) and homoscedasticity (Levene’s test). A two-way analysis of variance (ANOVA) was performed to identify statistical differences in transcript expression patterns across the experimental conditions for all genes independently, for each tissue. Post-hoc tests for multiple comparisons (Tukey tests) were applied whenever significant differences across treatments were observed. All statistical analyses were performed using a significance level of 0.05, using a custom python script and the program STATISTICA v.12 (StatSoft Inc., USA).

## Results

### Protein structural and functional evolution

**Fig 1:**
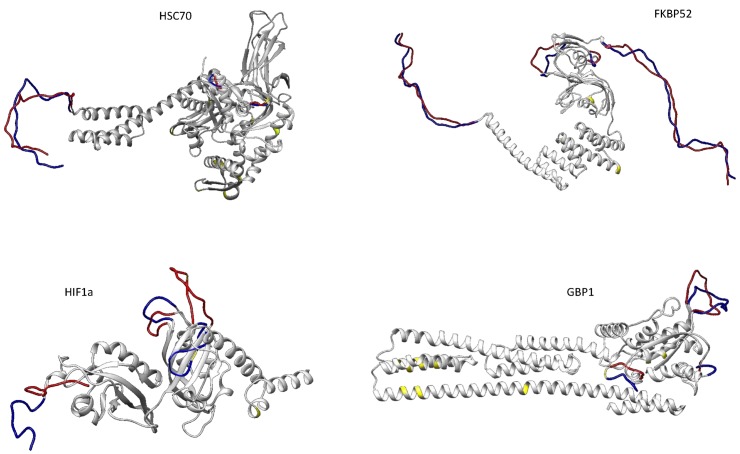
Structural differences between predicted proteins of the two species. Regions in light grey have no differences between species, blue and red indicate the conformation of *S*. *carolitertii* and *S*. *torgalensis* for that specific region and yellow represents the amino acids positions which correspond to non-synonymous substitutions.

Four of fourteen target proteins, showed alterations in their predicted tertiary structure between the two species (Fig 1), and two presented different predicted physical and chemical parameters (S4 Table, supporting information).

The physical and chemical parameters of the selected proteins were similar between species, with GBP1 and HSC70 presenting small changes in their theoretical isoelectric point (pI) and GBP1 and HSP90 having 1 unit differences in their respective aliphatic indexes (S4 Table, supporting information).

Regarding their tertiary structures, HSC70, FKBP52 (FK506-binding protein 4, encoded by the *fkbp4* gene), HIF1α and GBP1 showed differences between species. For HSC70, there were 11 noncontiguous aminoacids (a.a.) different between the two species (S5 Table, supporting information), but these did not coincide with the main predicted structural differences that are located in coil regions (Fig 1). FKBP52 had 3 non-synonymous substitutions (S5 Table, supporting information) that also did not overlap with observed structural changes, within coil regions, but instead were located mainly at termini, as observed for HSC70 (Fig 1).

In addition to the above-mentioned folding proteins, two other proteins presented structural alterations. HIF1α exhibited structural changes at the helix-loop-helix (bHLH), Per-ARNT-Sim (PAS) and DNA-binding domains. The HIF1α transcription factor presented two non-synonymous substitutions between species (S5 Table, supporting information), one of which overlaps with predicted structural changes in coil regions in the PAS domain (Fig 1 and S5 Table, supporting information).

The GBP1 protein presented 11 non-synonymous substitutions in the helical and globular protein domains (Fig 1 and S5 Table, supporting information). However, the locations of these altered amino acids did not coincide with the positions of structural changes observed in coil regions of the globular (GTP-binding) domain (Fig 1).

The remaining 10 predicted proteins presented no alterations between species (S1 Fig, supporting information).

### Gene expression

Stability values of the reference genes *pabpc1a*, *rpl35* and *rpsa* were high (less than 0.06 for all tissues and temperatures tested and, on average, less than 0.045) (S2 Fig, supporting information), with little variation (stability values of *rpsa* varied between 0.029 and 0.041, for *rpl35* between 0.017 and 0.051, and for *pabpc1a* between 0.030 and 0.059). These stability values are inferior to those observed by [49], which makes them suitable for gene expression normalization of our target genes.

**Fig 2:**
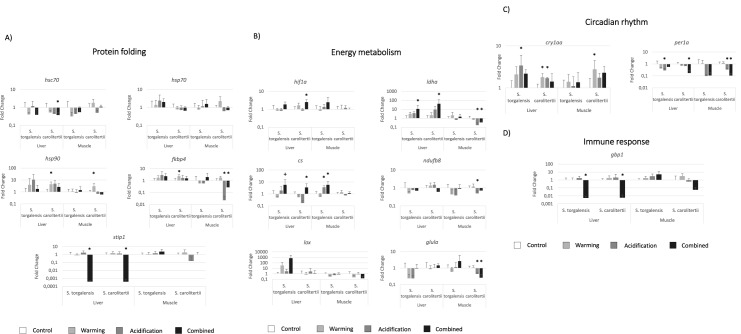
Gene expression of the genes involved in A) protein folding, B) energy metabolism, C) circadian rhythm and D) immune response. Gene expression values and significances are relative to the control condition. The * symbol represents a *p-value* < 0.05 and + symbol a 0.1 < *p-value* < 0.05 (and thus marginally significant).

Combined warming and acidification elicited the most significant changes in our genes of interest (in 11 genes for *S*. *carolitertii* and in 4 for *S*. *torgalensis*), followed by acidification (6 genes altered for *S*. *carolitertii* and 3 for *S*. *torgalensis*). Warming did not significantly alter *S*. *torgalensis* gene expression, but *S*. *carolitertii* presented significant differences in 5 genes (Fig 2).

Regarding differential expression of genes involved in protein folding, *S*. *carolitertii* presented significant changes in more genes, with differences between control and test conditions observed for *hsc70*, *hsp90aa1*, *fkbp4* and *stip1* (Fig 2A), while *S*. *torgalensis* only presented changes for *stip1* (Fig 2A). Most of the differences in observed gene expression were elicited by the warming and combined conditions, except for *fkbp4* for which a significant change under the acidification condition was observed in *S*. *carolitertii* muscle. No change was detected for the *hsp70* gene.

Regarding genes related to energy metabolism, most differences occurred under combined conditions of warming and acidification, with both species presenting several significant alterations (Fig 2B). The *ldha* and *cs* genes presented the greatest differences in expression, particularly for muscle tissue, in which distinct patterns were found for both species: *ldha* was downregulated in *S*. *carolitertii* and *cs* was upregulated in *S*. *torgalensis*. Both species showed a similar response in liver tissue, with both these genes being upregulated under combined conditions of warming and acidification (though for *cs* in *S*. *carolitertii* was only marginally significant). The *hif1a* gene was significantly upregulated in *S*. *torgalensis* liver under combined warming and acidification, presenting a similar pattern as that observed for the *cs* and *ldha* genes. However, only *hif1a* changes were statistically significant for *S*. *carolitertii* liver under the same combined conditions. Expression of the *ndufb8* and *glula* genes (Table 2) changed in *S*. *carolitertii* muscle, but that of the *lox* gene did not (Table 2).

Circadian clock genes (*cry1aa* and *per1a*) revealed significant changes under acidification for *S*. *torgalensis* liver tissue, whereas *S*. *carolitetii* presented significantly altered expression for these genes under all three conditions for liver and muscle tissues (Fig 2C).

The *gbp1* gene, which is involved in the immune response, presented major changes in fish exposed to the combined warming and acidification condition, being downregulated in the livers of both species (Fig 2D).

Significant (*p* < 0.05) synergistic effects between the combined factors of temperature and pH were observed in the liver for *S*. *carolitertii* (*hsp90aa1*, *fkbp4*, *stip1*, *cs*, *ndufb8* and *gbp1)* and *S*. *torgalensis* (*hsp90aa1*, *per1a* and *gbp1)*, as well as in the muscle for *S*. *carolitertii* (*lox)* and *S*. *torgalensis* (*ndufb8)*.

## Discussion

It is currently assumed that climate change, namely warming and acidification, will pose serious challenges to species survival and persistence [52]. In general, temperate species are potentially more adapted to deal with wide ranges of temperatures and pH on a seasonal and daily basis. To date, empirical data on the biological effects of warming and acidification on freshwater biota, especially endangered fish species, is scarce or still poorly understood [11,34]. To the best of our knowledge, our work represents the first comparative study integrating protein structural and functional analysis and gene expression changes in freshwater fish species exposed to experimental conditions of warming and acidification, simulating a future climate change scenario.

### Protein structural and functional evolution

First, we consider at the structural and functional evolution of 14 proteins in two Iberian endemic fish species (*S*. *carolitertii* from the North and *S*. *torgalensis* from the South). Of the 14 predicted proteins we studied, 3 proteins related to protein folding presented noticeable differences between species in either their physical and chemical parameters (HSP90) or in their structure (HSC70, FKBP52). Additionally, structural differences were found for the energy metabolism-related protein, HIF1α, and both functional and structural differences were found for GBP1, which is involved in the immune response.

We found that *S*. *torgalensis* displays a higher thermostability for HSP90. For HSC70, several structural changes between species were found in the coil regions of functional domains, which are of uncertain importance for its protein folding function [53,54]. The *fkbp4* gene encodes FK506-binding protein 4 (FKBP52), which possesses an N-terminal peptidylprolyl cis–trans isomerase domain (PPIase) and a C-terminal tetratricopeptide repeat domain (TPR). The PPIase domain is responsible for the cis-trans isomerization process that can limit this type of protein folding [55], whereas the TPR domain mediates protein–protein interactions. For example, FKBP52 interacts with HSP90, thereby facilitating the intracellular trafficking of steroid receptors. Moreover, this protein is involved in the regulation of interferon regulatory factor-4 and plays an important role in immunoregulatory gene expression in B and T lymphocytes [56]. Here, we observed alterations in both domains, suggesting that this protein has a potential role in climate change adaptation in these species. HIF1α is responsible for regulating many hypoxia-associated genes, as well as genes involved in glucose metabolism, cell proliferation and iron metabolism. Our predicted HIF1α proteins showed differences in all three functional domains, particularly in the DNA-binding domain that is crucial for the regulation of transcription [57]. However, changes in bHLH and PAS domains may interfere with protein-protein dimerization [57], which may be a key element in the regulatory activity of proteins such as enzymes, ion channels, receptors and transcription factors [58].

We also found structural changes between both species and a higher aliphatic index (thus higher thermostability) for *S*. *torgalensis* in the predicted GBP1 protein, which is induced by interferons and has antiviral activity [59,60]. The structural differences were mostly located in the GTP-binding domain of the protein, which hydrolyzes GTP to GDP, and is crucial for the function of the protein in antiviral defense [61].

The higher thermostability of HSP90 and GBP1 and the structural differences of GBP1 may indicate an advantage for *S*. *torgalensis* in a warmer environment. Additionally, the structural differences found between the two species for both species in HSC70, and HIF1α located in coil regions between functional domains have unclear impacts on protein function [53,54], even though these are particularly important regions for overall conformational flexibility [62]. These structural differences could be linked to the potential of this species to cope with warmer environments.

### Gene expression under future climate change scenario

Regarding gene expression, to date, only heat shock experiments had been conducted on these species [14,20]. In this study, we provide new clues as how these two species can acclimate to projected climate change by simulating the effects of increasing temperature and water acidity, both separately and combined. In general, the combination of both effects resulted in higher impacts on gene expression compared with the control condition. Although the resulting altered gene expression could be considered an additive effect of both conditions, for some genes, such as *stip1* and *gbp1* in the liver tissue of both species, the changes in expression were synergistic, since they were not observed in the independent temperature or pH experiments. Pimentel *et al*. (2015) observed cumulative changes in enzymatic activity under similar conditions (warming and acidification) in the flatfish *Solea senegalensis* [63]. Despite this, to date, many studies have focused on single stressors (e.g. either temperature or pH) [8,10,14,20]. Thus, our results emphasize the necessity to consider the combined effects of these stressors when assessing the impacts of climate change scenarios on organisms, since changes are neither the simple sum of these stressors nor can they be easily predicted by considering the effects of the two factors separately.

Across all experimental conditions, genes involved in protein folding presented differential expression only for *S*. *carolitertii*, with the exception of *stip1* that showed changes in both species. The heat shock proteins *hsc70* and *hsp90aa1* presented changes in quantitative gene expression for *S*. *carolitertii*, but *hsp70* did not. The differences in gene expression found for *hsc70*, support that structural differences between the two species can be important to protein function. Long-term changes in these genes may be disadvantageous since previous studies have shown that resources are reallocated from other crucial biological processes (e.g. growth) for the folding of denatured proteins [e.g. 10,59]. In previous studies, heat shock induced increased expression of both *hsp70* and *hsc70* as a response to acute thermal stress in *S*. *torgalensis* [14,20], probably to prevent protein denaturation [64,65]. However, in the present study, no change was observed for *hsp* genes in response to a milder temperature change for a longer period. Therefore, the fact that *S*. *torgalensis* have specific changes in protein structure at these genes, together with the fact that it coped with the new environment without major changes in the gene expression might indicate that this species has a higher thermal tolerance before eliciting stress responses.

HSP70 and HSP90 proteins usually form a complex of chaperones that help in the correct folding of important proteins for cell functioning. However, both proteins are capable of independent activity. While HSP70 is responsible for the folding of nascent proteins and other important cell processes (e.g. trafficking of proteins across membranes), the most common client proteins of HSP90 are regulators of transcription or protein kinases (see [66] for further details). Therefore, the observed differences in *hsp90aa1* gene expression may be related to subtsract interactions of HSP90 protein, with *S*. *carolitertii* possibly incurring altered transcriptional regulation under the warming condition.Moreover, these expression differences between the two species, in *hsp90aa1*, can be related to the higher thermostability of the corresponding coding protein observed in *S*. *torgalensis*. In contrast, pH *per se* did not affect the genes involved in protein folding, except for *fkbp4*, which possesses peptidylprolyl isomerase activity [56] and whose catalysis may depend on environmental pH [67]. Therefore, the lack of gene expression response in *S*. *torgalensis* could be related with the structural differences between the proteins of both species that encode this gene (FKBP52). Also, the observed results for *fkbp4* may be related with its immunoregulatory functions [56]. Stress-Induced Phosphoprotein 1 [*stip1* or *hop* (Hsp70-Hsp90 Organizing Protein)] mediates the transfer of proteins from HSP70 to HSP90, through the formation of an “intermediate complex” composed of these three proteins and the substrate protein (mainly steroid hormone receptors) [66]. The severe downregulation of *stip1* gene transcription under the combined warming and acidification condition in liver tissues of both species highlights the importance of synergistic effects in climate change studies.

The genes involved in energy metabolism presented an intricate and interconnected response (see S3 Fig, supporting information for a schematic representation). The transcription factor *hif1a* induces many genes during hypoxia, but also participates in other pathways such as glucose metabolism, with *ldha* being a target gene of this transcription factor [57,68]. In this study, we maintained the animals under normoxic conditions and an increase in transcription of both *hif1a* and *ldha* genes was observed in liver. Thus, the induction of *hif1a* gene expression seems to be more related to glucose metabolism rather than hypoxia. Importantly, the liver is capable of catabolism and anabolism at the same time; an ability not shared by any other organ or tissue [69].

However, gluconeogenesis is an expensive mechanism and we found that upregulation of the *ldha* gene was coupled with an increase in *cs* transcription, suggesting an increase in the usage of pyruvate by the citric acid cycle. Furthermore, in *S*. *torgalensis*, *ldha* expression in muscle was not altered between treatments, whereas *cs* was upregulated under acidic and combined conditions, suggesting a greater ability to produce ATP. However, *S*. *carolitertii* exhibited downregulation of *ldha* under the same conditions, with no significant change in *cs* transcription, so perhaps this species has a reduced capacity to produce ATP under the acidic and combined conditions. Also, the gene *ndufb8*, which encodes NADH dehydrogenase 1 beta subcomplex subunit 8, which is capable of independent respiratory chain activity in mitochondria [70], was downregulated in *S*. *carolitertii* under acidified conditions. Together, these results suggest that both species prioritize aerobic metabolism for energy production in muscle, with *S*. *torgalensis* showing a greater capability of producing energy under our experimental conditions compared to control by increasing the expression of *cs* and by maintaining *ldha* expression. Also, differences found in the expression of genes related with energy metabolism can result from the higher thermostability and structural differences found in HIF1α for *S*. *torgalensis*, since this protein is a main regulatory agent of this function.

Glutamine ammonia ligase or glutamine synthetase (encoded by the *glula* gene) plays a key role in nitrogen metabolism, catalyzing the conversion of ammonia and glutamate to glutamine, a less toxic compound that is used in the production of several other metabolites [71]. We only observed differential expression under acidification alone, with warming having little or no significant effect. Thus, the catalytic activity of this enzyme may decrease at lower pH in *S*. *carolitertii* muscle. Though we did not feed our experimental groups of fish differently, demand for nitrogen compounds is expected to decrease under increased temperatures and so herbivory is increased, which has been reported in omnivorous copepods and fish [72,73]. Therefore, the *glula* gene might be a suitable biomarker for the usage of nitrogen in omnivorous fish undergoing climate change.

We did not find significant differences for the *lox* gene among fish under different conditions. Lysyl oxidase (LOX) catalyzes the formation of lysine-derived cross-links in collagen and elastin and it is involved in several other biological functions (e.g. development, tumor suppression, cell motility and cellular senescence) [74]. Therefore, the absence of differences may be due to the fact that the *lox* gene is vital during development and in atypical cell functioning [74].

Warming and acidification have impacts on many biological processes that occur in vertebrate cells. Despite limited evidence that circadian clock genes may be directly impacted by climate change, temperature may trigger the responses of such genes [75, 76], as observed for the species in this study [20]. We found that the two circadian clock genes we studied, *cry1aa* and *per1a*, presented significant changes under both warming and acidification conditions. Expression was increased for *cry1aa* and decreased for *per1a* in both species. The *cry1aa* gene is known to be induced in fish during the early morning, whereas *per1a* has higher expression late at night (end of the dark period) [77]. Contrary to *cry1aa*, *per1a* gene does not exhibit light-dependent expression [77]. Therefore, disruption of this balance in the circadian clock of fish may have profound effects on fish metabolism and behavior (such as feeding and mating behavior), particularly given that the changes were not the result of experimental changes in photoperiod. For a more detailed mechanistic explanation on this subject, a study of all genes involved in the circadian clock would aid our understanding of the regulation of the pool of *cry* and *per* genes that are involved in clock regulation.

There is growing concern about the effects of environmental change on the immune system of vertebrates [7,10]. Some evidence that temperature may alter gene expression of immune response-related genes is already available [10,20,78]. Our results, raise some concerns for medium- to long-term exposure to predicted climate change, since a drastic downregulation was observed for the *gpb1* gene for the combined warming and acidification condition. Although we analyzed only one gene related to the immune system, the combination of these two environmental factors severely decreased its expression, putatively leading to its suppression. Therefore, further attention should be paid to the effects and interactions of the multiple environmental factors involved in climate change on genes involved in the immune response.

## Conclusions

Climate change projections for freshwater ecosystems are scarce and may be worse than we simulated here, particularly for the acidification of these ecosystems, where organic matter content may be extremely variable between water bodies and seasons, contrary to what is observed in oceanic waters [34,41]. In this study, we examined differences in protein configuration and in gene expression between two endemic Iberian freshwater fish species that inhabit different climatic regions, *S*. *carolitertii* in the Atlantic-type northern region and *S*. *torgalensis* in the Mediterranean-type southwestern region. We observed protein structural differences between the two species for HSC70, FKBP52 and HIF1α and higher thermostability for HSP90 and GBP1 in *S*. *torgalensis*. Most of the changes in gene expression were observed for *S*. *carolitertii*, whereas *S*. *torgalensis* showed no major changes in the heat shock response or in respiratory capacity. Taken together, these results suggest that *S*. *torgalensis*, which lives in a warmer environment, is less impacted by temperature increases and acidification. Consequently, our results suggest that *S*. *torgalensis* could be capable of dealing with the IPCC projections of warming and/or acidification at the end of this century. Our study highlights the importance of assessing the potential of endangered freshwater species to cope with projected climate change conditions for the proper implementation of conservation strategies.

## Supporting information

S1 Fig Protein structure predictions for proteins with minor or no differences between species. Regions in light grey have no differences between species, blue and red indicate the conformation of *S*. *carolitertii* and *S*. *torgalensis* for that specific region and yellow represents the amino acids which correspond to non-synonymous substitutions.
(PDF)

S2 Fig Stability values calculated for the reference genes (*rpsa*, *rpl35* and *pabpc1a*), showing their overall stability and for each organ and condition analyzed. The lower the stability value the better the reference gene and thus less variable across the experimental conditions.
(PDF)

S3 Fig Schematic representation of the pathways discussed in this research for the genes involved in energy metabolism. Doted arrows indicate gene expression regulation from the source to the sink gene; dashed arrows represent a source gene that encodes a protein is responsible for substrate conversion; and full arrows indicate a direct conversion. Target genes are represented with squares, except for *hif1a* (represented with a rectangle with two curved sides), which is a key gene in the regulation of many gene involved in these pathways. Circles indicate genes which regulate relevant pathways but that are not target genes and polygons symbolize the substrates.
(PDF)

S1 Table Primer pairs used to re-sequence genes in Sanger with their PCR amplification conditions. (DOCX)

S2 Table Real-time RT-PCR primer pairs for reference and target genes and their efficiency values calculated in LinRegPCR (Ruijter *et al*., 2009). Real-time PCRs were done in a final volume of 10 μL, containing 5 μL of Sso Advanced universal SYBR® Green supermix (2x) (Bio- Rad, Hercules, CA, USA) and 0.4 μL of each primer (with a concentration of 0.4 μM). The assay conditions included an initial denaturation step at 95°C for 30 s, followed by 40 cycles at 95°C for 10 s and 60°C for 30 s.
(DOCX)

S3 Table Gene expression values of reference and target genes in the transcriptomes of both species described in Jesus *et al*. (2016). Reference genes have a column for the differential gene expression value between *S*. *pyrenaicus* males and females from (Genomic resources development consortium *et al*., 2015b). Non-DE and N/A stands for genes that are not significantly differentially expression and not applicable, respectively.
(DOCX)

S4 Table Predicted proteins physical and chemical parameters. (XLSX)

S5 Table Non-synonymous substitutions for the translated predicted protein structures (in a.a.). (DOCX)
