# Supplementary material for: Protein analysis and gene expression indicate differential vulnerability of Iberian fish species under a climate change scenario
Source: PLoS One. 2017 Jul 18;12(7):e0181325. doi: 10.1371/journal.pone.0181325 (PMC5515415; doi:10.1371/journal.pone.0181325)

Figure S1

Protein folding

STIP1

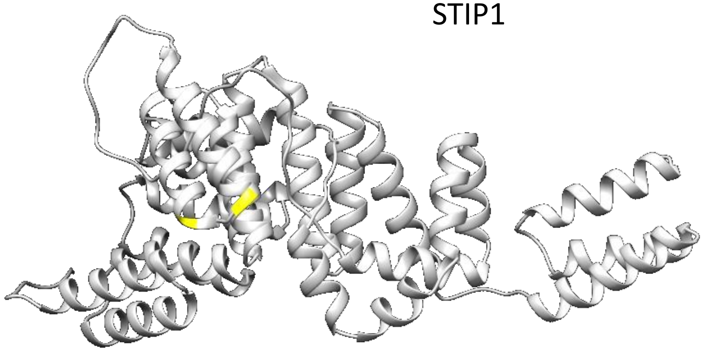

HSP70

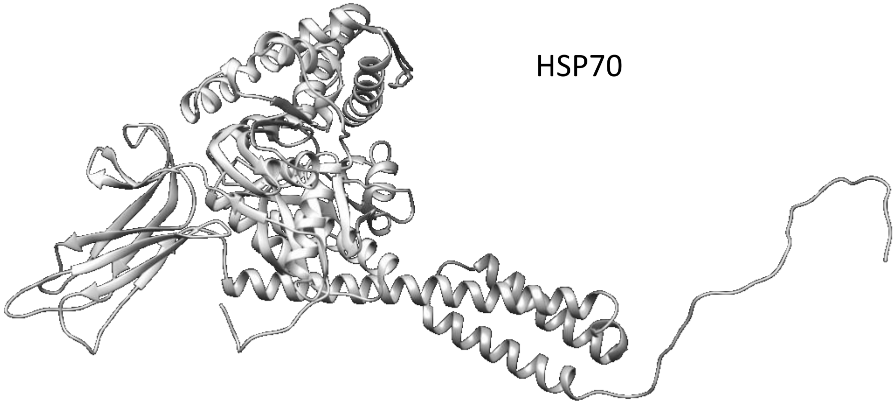

HSP90

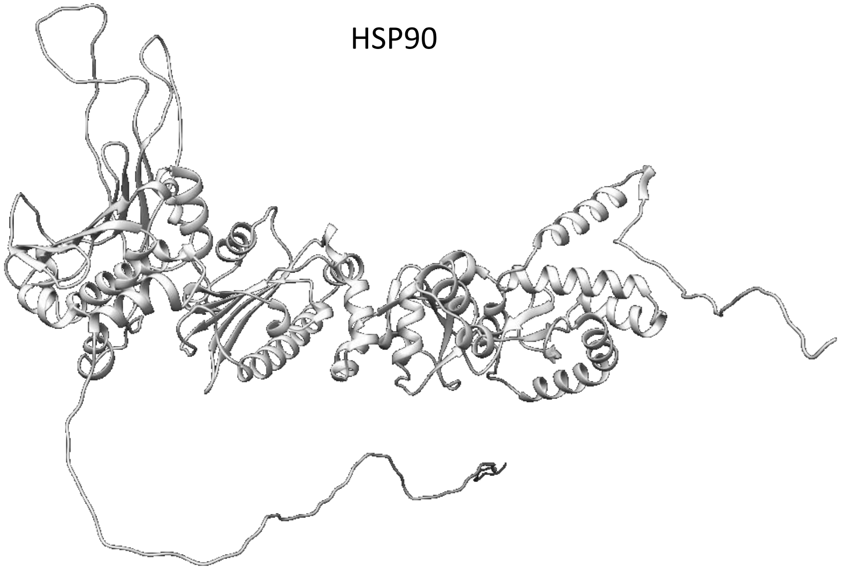

Energy metabolism

Figure S1

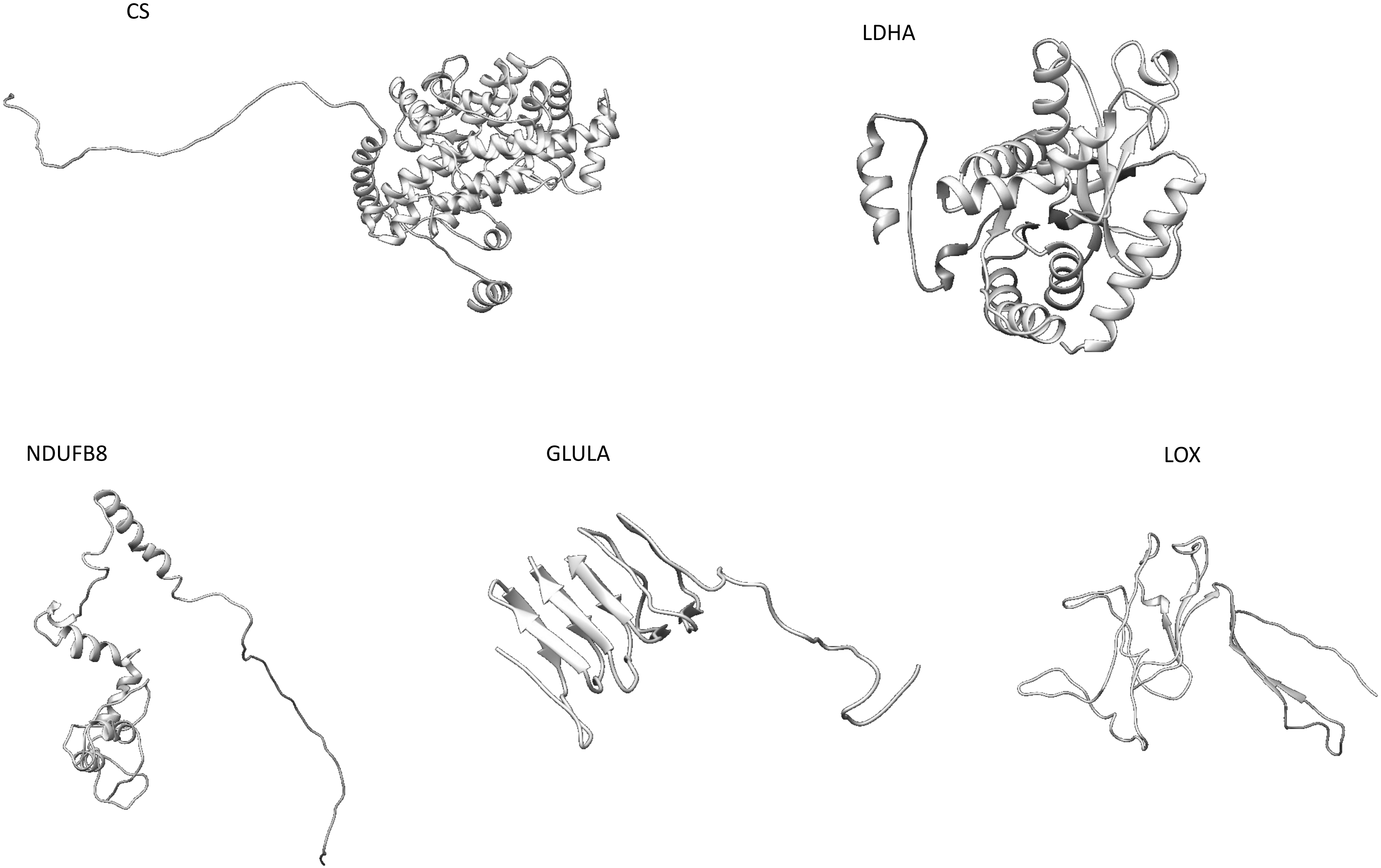

Figure S1

Cyrcadian rhythm

PER1A

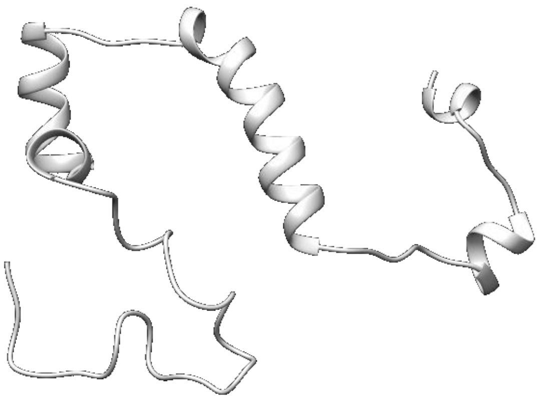

CRY1AA

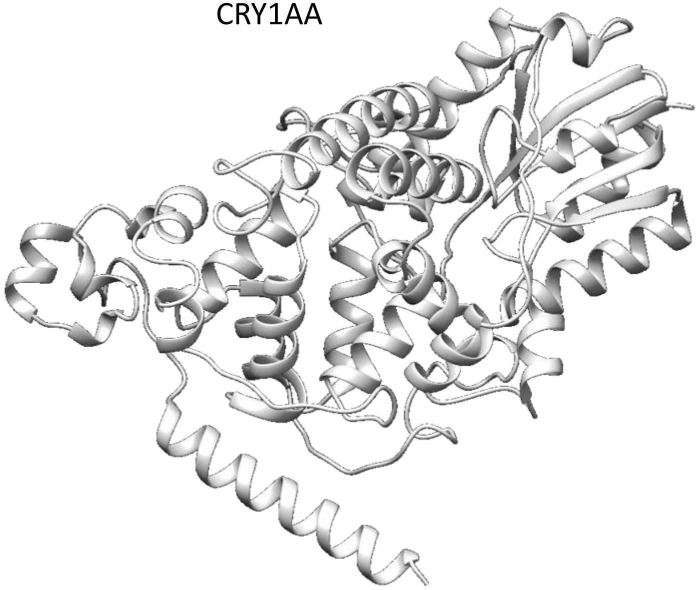

Supplement: S1 Fig — Regions in light grey have no differences between species, blue and red indicate the conformation of S. carolitertii and S. torgalensis for that specific region and yellow represents the amino acids which correspond to non-synonymous substitutions. (PDF) [file pone.0181325.s001.pdf]
