# Supplementary figures and images for: Protein analysis and gene expression indicate differential vulnerability of Iberian fish species under a climate change scenario
Source: PLoS One. 2017 Jul 18;12(7):e0181325. doi: 10.1371/journal.pone.0181325 (PMC5515415; doi:10.1371/journal.pone.0181325)

Figure S2

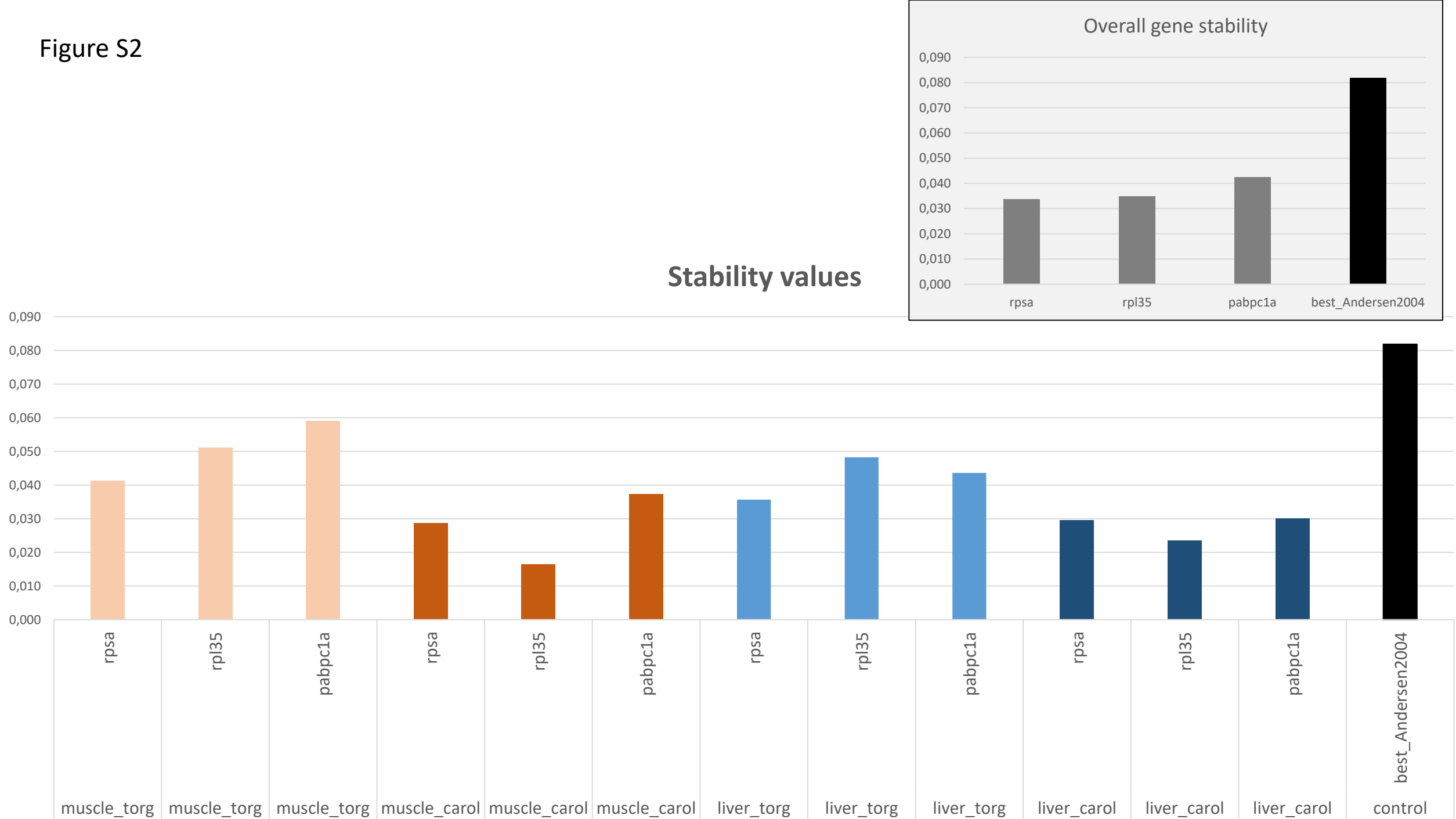

Supplement: S2 Fig — The lower the stability value the better the reference gene and thus less variable across the experimental conditions. (PDF) [file pone.0181325.s002.pdf]

Figure S3

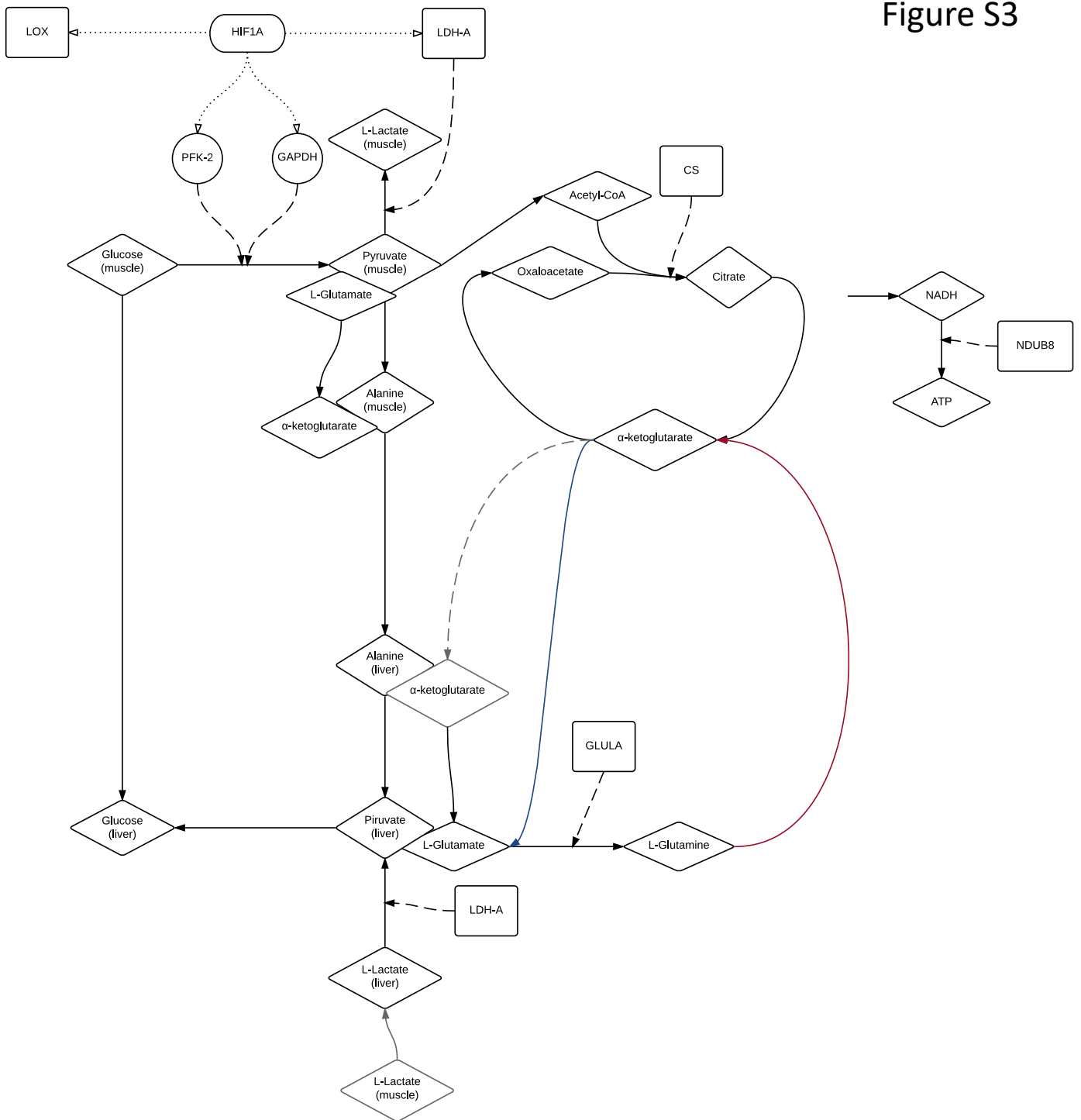

Supplement: S3 Fig — Doted arrows indicate gene expression regulation from the source to the sink gene; dashed arrows represent a source gene that encodes a protein is responsible for substrate conversion; and full arrows indicate a direct conversion. Target genes are represented with squares, except for hif1a (represented with a rectangle with two curved sides), which is a key gene in the regulation of many gene involved in these pathways. Circles indicate genes which regulate relevant pathways but that are not target genes and polygons symbolize the substrates. (PDF) [file pone.0181325.s003.pdf]
