# Supplementary material for: Protein analysis and gene expression indicate differential vulnerability of Iberian fish species under a climate change scenario
Source: PLoS One. 2017 Jul 18;12(7):e0181325. doi: 10.1371/journal.pone.0181325 (PMC5515415; doi:10.1371/journal.pone.0181325)
Supplement: S1 Table — (DOCX) [file pone.0181325.s004.docx]

**Supplementary Tables S1** - Primer pairs used to re-sequence genes in Sanger with their PCR amplification conditions.

| Genes | Primer names | Primer Sequence |
| --- | --- | --- |
| *ldha* (1) | ldha_f1 | Forward: 5' - GCTGAAAGGAGAGGTTATGG - 3' |
|  | ldha_r1 | Reverse: 5' - AATGGTTAGAGGCAGTGAGG - 3' |
| *ldha* (2) | ldha(2)_fw | Forward: 5' - GACCTGTTAGCCAATAGACC -3' |
|  | ldha(2)_rv | Reverse: 5' - TCCAGCTGATACACAAAGTG - 3' |
| *cry1a* (1) | cry1a_fw | Forward: 5' - TCTTCCAGCAGTTCTTCCAC - 3' |
|  | cry1a_rv | Reverse: 5' - TGTGCAGATTACAGAGCCAG - 3' |
| *cry1a* (2) | cry1a(2)_fw | Forward: 5' - CACGGCAGGATGGTTTAC - 3' |
|  | cry1a(2)_rv | Reverse: 5' - TGTGCAGATTACAGAGCC - 3' |
| *gbp1* | gbp1_fw | Forward: 5' - GAAGTCCTACCTTATGAACC -3' |
|  | gbp1_rv | Reverse: 5' - ATGCTTACAGCTTCCTCCAG -3' |
| *hif1a* | hif1a_fw | Forward: 5' - GAGTCCGAGGTGTTCTACGAG - 3' |
|  | hif1a_rv | Reverse: 5' - GCTCTGTCATGGTCTGCTGC - 3 |
| *hsc70* | hsc70_fw | Forward: 5' - GACCTTCACCACTTACTCAG - 3' |
|  | hsc70_rv | Reverse: 5' - CACTTCCTCAATGGTAGGAC - 3' |
| *fkbp4* | fkbp4_fw | Forward: 5' - CGCAGGATCATCACTAAGG - 3' |
|  | fkbp4_rv | Reverse: 5' - CATGCCATTATGCTGCAGTT - 3' |
| *hsp90* | hsp90_fw | Forward: 5' - GCTTTCCCTCAAGGACTACG -3' |
|  | hsp90_rv | Reverse: 5' - GGTTGAGTAATGTCCTCCACAG - 3' |

|  |  |  |  |  |  |  |  |  |  |  |  |
| --- | --- | --- | --- | --- | --- | --- | --- | --- | --- | --- | --- |
|  |  |  | PCR Cycles | | | | | | |  |  |
|  | Initial Denaturation | | Denaturation | | Annealing | | Extension | |  | Final Extension | |
| Genes | Temp (ºC) | Time (s) | Temp (ºC) | Time (s) | Temp (ºC) | Time (s) | Temp (ºC) | Time (s) | Cycles | Temp (ºC) | Time (s) |
| *ldha* (1) | 95 | 300 | 95 | 60 | 60 | 60 | 72 | 60 | 35 | 72 | 600 |
| *ldha* (2) | 95 | 300 | 95 | 60 | 54 | 60 | 72 | 60 | 35 | 72 | 600 |
| *cry1a* (1) | 95 | 300 | 95 | 60 | 56 | 60 | 72 | 60 | 35 | 72 | 600 |
| *cry1a* (2) | 95 | 300 | 95 | 60 | 56 | 60 | 72 | 60 | 35 | 72 | 600 |
| *gbp1* | 94 | 300 | 95 | 45 | 56 | 60 | 72 | 60 | 35 | 72 | 600 |
| *hif1a* | 95 | 300 | 95 | 60 | 60 | 60 | 72 | 60 | 35 | 72 | 600 |
| *hsc70* | 95 | 300 | 95 | 60 | 56 | 60 | 72 | 60 | 35 | 72 | 600 |
| *fkbp4* | 95 | 300 | 95 | 60 | 58 | 60 | 72 | 60 | 35 | 72 | 600 |
| *hsp90* | 95 | 300 | 95 | 60 | 52 | 60 | 72 | 60 | 35 | 72 | 600 |

| Genes | Taq Buffer (5x) | MgCl_2_ (10 mM) | dNTP's (10 mM) (2 mM each dNTP) | Primers (10 µM) | Taq (5U/µL) |
| --- | --- | --- | --- | --- | --- |
| *ldha* (1) | 5 | 2 | 2,5 | 0,75 | 0,12 |
| *ldha* (2) | 5 | 2 | 2,5 | 0,75 | 0,10 |
| *cry1a* (1) | 5 | 2 | 2,5 | 0,75 | 0,12 |
| *cry1a* (2) | 5 | 2 | 2,5 | 0,75 | 0,12 |
| *gbp1* | 5 | 2 | 2,5 | 0,75 | 0,15 |
| *hif1a* | 5 | 2 | 2,5 | 0,75 | 0,12 |
| *hsc70* | 5 | 1,5 | 2,5 | 0,75 | 0,12 |
| *fkbp4* | 5 | 2 | 2,5 | 0,75 | 0,15 |
| *hsp90* | 5 | 2 | 2,5 | 0,75 | 0,12 |
