# Supplementary material for: Protein analysis and gene expression indicate differential vulnerability of Iberian fish species under a climate change scenario
Source: PLoS One. 2017 Jul 18;12(7):e0181325. doi: 10.1371/journal.pone.0181325 (PMC5515415; doi:10.1371/journal.pone.0181325)
Supplement: S2 Table — Real-time PCRs were done in a final volume of 10 μL, containing 5 μL of Sso Advanced universal SYBR® Green supermix (2x) (Bio- Rad, Hercules, CA, USA) and 0.4 μL of each primer (with a concentration of 0.4 μM). The assay conditions included an initial denaturation step at 95°C for 30 s, followed by 40 cycles at 95°C for 10 s and 60°C for 30 s. (DOCX) [file pone.0181325.s005.docx]

**Suplementary Table S2** - Real-time RT-PCR primer pairs for reference and target genes and their efficiency values calculated in LinRegPCR (Ruijter et al., 2009). Real-time PCRs were done in a final volume of 10 µL, containing 5 µL of Sso Advanced universal SYBR® Green supermix (2x) (Bio- Rad, Hercules, CA, USA) and 0.4 µL of each primer (with a concentration of 0.4 µM). The assay conditions included an initial denaturation step at 95 °C for 30 s, followed by 40 cycles at 95 °C for 10 s and 60 °C for 30 s.

| Gene name | Primers | | Efficiency (%) | References |
| --- | --- | --- | --- | --- |
|  | forward | reverse |  |  |
| pabpc1a | 5' - GCAAAGTGTTCGTCGGTC - 3' | 5' - CTCGTCATCCATATCCTCTCC - 3' | 97,06 | N/A |
| rpl35 | 5' - CAAGCCTTTGGACCTGAGG - 3' | 5' - GGTTCTCCTCGTGTTTGGTCA - 3' | 96,56 | N/A |
| rpsa | 5' - CATCCCAACCATTGCCCT - 3' | 5' - TCCACCACATCAGACCCA - 3' | 96,54 | N/A |
| *cry1a* | 5' - CCTTCTTCCAGCAGTTCTTC - 3' | 5' - GTATGTAGTCTCCGTTGGG - 3' | 97,22 | N/A |
| *cs* | 5' - CTGTTGCCCAAAGCTTCCG - 3' | 5' - GCCCACTCCTTAGACAACCA - 3' | 94,38 | N/A |
| *fkbp4* | 5' - AATCCCACCCAACGCTACC - 3' | 5' - CACACTTCCACAGATGCACC - 3' | 97,21 | N/A |
| *gpb1* | 5' - GAAGTCCTACCTTATGAACCGC - 3' | 5' - CCAGCCGTCATTCTTAGAGTC - 3' | 96,96 | N/A |
| *glula* | 5' - CCAGTCAGTCTACGAGCA - 3' | 5' - GCCACACTAACTTTAGCACC - 3' | 97,38 | N/A |
| *hif1a* | 5' - CCTCATCCCTCAAACATCG - 3' | 5' - GGCTCATATCCCATCAGC - 3' | 97,24 | N/A |
| *hsc70* | 5' - TTTGCTGTTGGATGTCACTC - 3' | 5' - GTGGGAATGGTGGTGTTC - 3' | 96,92 | Jesus*et al.*2013 |
| *hsp70* | 5' - AATTCCACCTGCACCACG - 3' | 5' - TCTCCTCTTTGCTCAGTCTG - 3' | 97,47 | Jesus*et al.*2013 |
| *hsp90* | 5' - CTGTTTATTCCCAGAAGAGCCTCC - 3' | 5' - TGTCCATGATAAAGACCCTGCG - 3' | 96,65 | N/A |
| *ldha* | 5' - TCTGACTGACGAACTCGCC - 3' | 5' - TCCAGCAGTCACAACCACC - 3' | 96,04 | N/A |
| *lox* | 5' - ACCAGATACTTCCAGAACGGT - 3' | 5' - GAACCTCAGCAGAACCCT - 3' | 96,32 | N/A |
| *nkx3.2* | 5' - CCGTTCTCCATTCAAGCCA - 3' | 5' - TGTCGTTGTCCTCGCTCAG - 3' | 97,65 | N/A |
| *nudb8* | 5' - GAAGATTACCAGCCCTTTCC - 3' | 5' - CGTGTCAACCCTATTCCTG - 3' | 96,65 | N/A |
| *per1a* | 5' - GAGTTAACGCAGGTCCAC - 3' | 5' - GGAGGAGTCAAGAAATCTGG - 3' | 97,41 | N/A |
| *stip1* | 5' - GCCTTAGACCCTTCCAATCAC - 3' | 5' - AGTCGCCCAAGAAACTCC - 3' | 97,01 | N/A |
