# Supplementary material for: Protein analysis and gene expression indicate differential vulnerability of Iberian fish species under a climate change scenario
Source: PLoS One. 2017 Jul 18;12(7):e0181325. doi: 10.1371/journal.pone.0181325 (PMC5515415; doi:10.1371/journal.pone.0181325)
Supplement: S3 Table — Reference genes have a column for the differential gene expression value between S. pyrenaicus males and females from (Genomic resources development consortium et al., 2015b). Non-DE and N/A stands for genes that are not significantly differentially expression and not applicable, respectively. (DOCX) [file pone.0181325.s006.docx]

**Suplementary Table S3** - Real-time RT-PCR primer pairs for reference and target genes and their efficiency values calculated in LinRegPCR (Ruijter et al., 2009). Real-time PCRs were done in a final volume of 10 µL, containing 5 µL of Sso Advanced universal SYBR® Green supermix (2x) (Bio- Rad, Hercules, CA, USA) and 0.4 µL of each primer (with a concentration of 0.4 µM). The assay conditions included an initial denaturation step at 95 °C for 30 s, followed by 40 cycles at 95 °C for 10 s and 60 °C for 30 s.

| gene name | *S. carolitertii* | | | *S. torgalensis* | | | *S. pyrenaicus* | GO description | Functional category |
| --- | --- | --- | --- | --- | --- | --- | --- | --- | --- |
|  | Fins | Liver | Muscle | Fins | Liver | Muscle | Brain* |  |  |
| *rpsa* | -0,24 | -0,29 | -0,21 | 0,34 | -0,11 | 0,36 | -0,27 | N/A | N/A |
| *rpl35* | -0,14 | -0,02 | -0,58 | 0,10 | 0,00 | 0,28 | -0,26 | N/A | N/A |
| *pabpc1a* | -0,01 | -0,10 | -0,03 | -0,09 | -0,47 | -0,45 | -0,24 | N/A | N/A |
| *per1a* | non DE | non DE | non DE | -3,39 | non DE | -7,47 | N/A | response to oxidative stress | circadian rhythm |
| *cry1a* | non DE | non DE | non DE | -10,70 | non DE | -9,08 | N/A | response to oxidative stress | circadian rhythm |
| *hsc70* | non DE | 4,33 | -0,77 | non DE | non DE | -9,72 | N/A | protein folding | protein folding |
| *hsp70* | 9,41 | 7,40 | 18,49 | 16,54 | 18,31 | 20,07 | N/A | protein folding | protein folding |
| *hsp90* | 8,21 | 8,18 | 5,59 | 8,87 | 4,24 | 4,69 | N/A | protein folding | protein folding |
| *stip1* | non DE | non DE | non DE | 3,84 | 5,75 | 9,35 | N/A | protein folding | protein folding |
| *fkbp4* | non DE | non DE | non DE | non DE | 3,40 | 12,09 | N/A | protein folding | protein folding |
| *hif1a* | non DE | non DE | -0,73 | non DE | -1,38 | 0,99 | N/A | response to oxidative stress | energy metabolism |
| *ldha* | non DE | non DE | non DE | non DE | non DE | -6,38 | N/A | response to oxidative stress | energy metabolism |
| *cs* | non DE | non DE | non DE | 0,93 | -0,70 | non DE | N/A | response to oxidative stress | energy metabolism |
| *ndub8* | non DE | non DE | -4,26 | non DE | non DE | 13,10 | N/A | response to oxidative stress | energy metabolism |
| *glula* | non DE | non DE | 2,23 | non DE | non DE | -6,61 | N/A | response to oxidative stress | energy metabolism |
| *lox* | non DE | non DE | 7,30 | non DE | non DE | -8,65 | N/A | skeletal system development | energy metabolism |
| *gbp1* | non DE | non DE | 7,06 | non DE | non DE | -4,36 | N/A | immune response | immune response |
|  |  |  |  |  |  |  |  |  |  |
