# Supplementary material for: Protein analysis and gene expression indicate differential vulnerability of Iberian fish species under a climate change scenario
Source: PLoS One. 2017 Jul 18;12(7):e0181325. doi: 10.1371/journal.pone.0181325 (PMC5515415; doi:10.1371/journal.pone.0181325)
Supplement: S5 Table — (DOCX) [file pone.0181325.s008.docx]

**Supplementary Table S5** - Non synonymous substitutions for the translated predicted protein structures (in a.a.).

| Functional category | gene | residues with non synonymous | raptor model coverage | raptor template Id |
| --- | --- | --- | --- | --- |
| protein folding | *hsp70* | N/A | 100%/100% | 5E84 |
|  | *hsc70* | 20;47; 81; 191; 243; 249; 254; 255; 288; 291; 315 | 100%/100% | 5E84 |
|  | *hsp90* | 741; 744; 745 | 100%/93% | 2CG9 |
|  | *fkbp4* | 222; 323; 351 | 100%/100% | 1kt1 |
|  | *stip1* | 244; 248 | 100%/100% | 1elw |
| energy metabolism | *hif1a* | 44; 200 | 46%/46% | 4zp4 |
|  | *ldha* | N/A | 100%/100% | 1v6a |
|  | *cs* | N/A | 100%/100% | 2cts |
|  | *ndufb8* | N/A | 100%/100% | 1t7n |
|  | *glula* | 4; 10 | 100%/100% | 4wa0 |
|  | *lox* | N/A | 43%/43% | 3ob8 |
| circadian rythm | *cry1a* |  | 80%/80% | 4ct0 |
|  | *per1a* | 97 | 59% | 4ct0 |
| immune system | *gbp1* | 54; 57; 65; 343; 345; 348; 352; 399; 403; 407; 430 | 100%/100% | 1dg3 |
